# Simultaneous 3D Construction and Imaging of Plant Cells Using Plasmonic Nanoprobe-Assisted Multimodal Nonlinear Optical Microscopy

**DOI:** 10.3390/nano13192626

**Published:** 2023-09-23

**Authors:** Kun Liu, Yutian Lei, Dawei Li

**Affiliations:** 1 School of Optoelectronic Engineering and Instrumentation Science, Dalian University of Technology, Dalian 116024, China; 2 Department of Civil Engineering, University of Nebraska-Lincoln, Lincoln, NE 68588, USA

**Keywords:** multimodal nonlinear optical imaging, plant cells, femtosecond laser, noble metal ions, plasmonic nanostructures, 3D cellular construction

## Abstract

Nonlinear optical (NLO) imaging has emerged as a promising plant cell imaging technique due to its large optical penetration, inherent 3D spatial resolution, and reduced photodamage; exogenous nanoprobes are usually needed for nonsignal target cell analysis. Here, we report in vivo, simultaneous 3D labeling and imaging of potato cell structures using plasmonic nanoprobe-assisted multimodal NLO microscopy. Experimental results show that the complete cell structure can be imaged via the combination of second-harmonic generation (SHG) and two-photon luminescence (TPL) when noble metal silver or gold ions are added. In contrast, without the noble metal ion solution, no NLO signals from the cell wall were acquired. The mechanism can be attributed to noble metal nanoprobes with strong nonlinear optical responses formed along the cell walls via a femtosecond laser scan. During the SHG-TPL imaging process, noble metal ions that crossed the cell wall were rapidly reduced to plasmonic nanoparticles with the fs laser and selectively anchored onto both sides of the cell wall, thereby leading to simultaneous 3D labeling and imaging of the potato cells. Compared with the traditional labeling technique that needs in vitro nanoprobe fabrication and cell labeling, our approach allows for one-step, in vivo labeling of plant cells, thus providing a rapid, cost-effective method for cellular structure construction and imaging.

## 1. Introduction

Nonlinear optics (NLO), including multiphoton luminescence, second-harmonic generation (SHG), third-harmonic generation (THG), four-wave mixing (FWM) and coherent anti-stokes Raman scattering (CARS), have been widely applied in the biological fields since their inception [1,2,3,4,5,6,7,8,9] Unlike linear optical microscopy, NLO microscopy offers inherent three-dimensional (3D) spatial resolution, relatively large optical penetration into tissues, and reduced photodamage [10,11] which allows for the viewing of cells that are living and functioning. In most cases, more than one NLO microscopies are needed for biological studies since different organic components may have different NLO responses. The multimodal NLO imaging system has offered an approach for comparing multi-images from different quantum processes, providing rich information for the study of various biological systems [12,13,14].

On the other hand, although many ordered structures in cells and tissues can produce inherent NLO signals, exogenous probes are frequently used to increase the contrast or to selectively image nonsignal targets. For example, exogenous SHG probes, including SHG dye or SHG nanoprobes, can be employed to extend their application to nonsignal targets such as the lipid bilayer in cells [15]. Although dyes have been extensively investigated for use as SHG probes, they suffer from photobleaching. Therefore, an active area of interest for researchers is the development of novel nanoprobes that are less prone to bleaching and blinking, while maintaining strong NLO responses [16,17,18,19].

Compared with normal dye probes, noble metal nanoprobes are photostable, non-photobleaching, and biocompatible. Considerable research has proved that noble metal nanostructures such as silver and gold nanoparticles (NPs) exhibit various NLO properties, including two-photon luminescence (TPL) [20], SHG [21,22], THG [23] and FWM [24], thus enabling them to be used as nanoprobes for labeling and imaging [10]. Traditionally, noble metal nanoprobes must be fabricated externally and then transported inside the cell through functional groups or via a bonded antibody. Moreover, the size of the noble metal NPs must be well controlled in order to pass through the cell membranes [25] which makes the labeling process complicated, challenging, and laborious.

As we know, plant cell walls are mostly composed of cellulose, which are invisible to conventional NLO imaging techniques. In this work, we report for the first time an innovative approach for in vivo and simultaneous 3D labeling and imaging of a complete potato cell structure using plasmonic nanoprobe-assisted multimodal NLO microscopy. Instead of fabricating noble metal NPs outside the cell and then transporting exogenous NPs into the cells, we added a noble metal ion solution to the cells directly during the femtosecond (fs) laser imaging process. The results show that noble metal ions can cross the cell membrane easily through osmotic pressure and were rapidly reduced to plasmonic NPs in situ via a fs laser scan. The as-grown noble metal NPs were found to exhibit strong second-order (TPL, SHG) and third-order (FWM) NLO responses. More interestingly, these noble metal nanoprobes were selectively anchored onto the cell walls. With this approach, the whole structure of potato cells can be simultaneously labeled and imaged. Compared with traditional labeling techniques, our approach offers a simple and facile way for in vivo construction and the 3D imaging of plant cells.

## 2. Materials and Methods

Reagents and materials. Silver nitrate (purity > 99%), gold (III) chloride trihydrate (>99.9% trace metal basis) were purchased from Sigma-Aldrich (St. Louis, MO, USA) and used without further purification. Deionized water with an electrical resistivity of 18 MΩ-cm was used for preparing the silver ion and gold ion solutions with a concentration of 0.3 mM and 3.7 mM, respectively. Fresh potatoes were cut into thin slices and fixed onto glass slides.

Multimodal nonlinear optical imaging of potato cells. The nonlinear optical properties of as-prepared and noble metal NPs-decorated potato cell samples were investigated using a home-made multiphoton nonlinear optical microscopy system. A commercial Ti:Sapphire fs laser with a center wavelength of 800 nm was used as the laser source. Combined with a supercontinuum generator, two incident laser beams with tunable powers were generated. One was called a pump laser beam, which was formed via introducing an 800 nm laser beam through an attenuator and a delay line. The other was called a probe laser, which was formed via a 500 mW laser beam to generate the supercontinuum which was then filtered through a long-pass filter. The pump-probe laser beams were then collinearly focused onto the sample surfaces. A spectrometer was used for the nonlinear optical spectra measurement. The nonlinear optical imaging signals were collected using PMTs. With the application of a 390/40 nm emission filter, a 495–540 nm band-pass filter, and a 647/57 nm band-pass filter before PMTs, three different nonlinear optical images were simultaneously acquired.

Fs laser-induced formation of noble metal nanoparticles. First, silver (gold) ion solutions were prepared through dissolving silver nitrate (gold chloride trihydrate) in deionized water. Second, the samples such as the fused silica substrates were immersed into a noble metal ion solution. Third, an 800 nm fs laser beam was focused onto the sample surface for irradiation and/or scanning. After fs laser irradiation of a single point, noble metal nanoparticles were formed in a short time (Figure 4b) and nanoparticle aggregates were formed with a long-time irradiation (Figure 4c); after fs laser scanning once in a particular area, a uniform noble metal nanoparticle film was formed (Figure 4f).

Other characterizations. Raman analyses were performed at room temperature in a micro-Raman spectrometer (Renishaw InVia plus, Renishaw, Gloucestershire, UK). Raman spectra and mapping were collected through a 50× objective lens with an exposure time of 10 s and 1 s, respectively, at each position. Morphological characterization of the potato cells and the noble metal particles was carried out using field emission scanning electron microscopy (SEM, JSM-7600F, JEOL Ltd., Tokyo, Japan).

## 3. Results and Discussion

### 3.1. Characterization of Potato Cells Using Common Imaging Techniques

**Figure 1:**
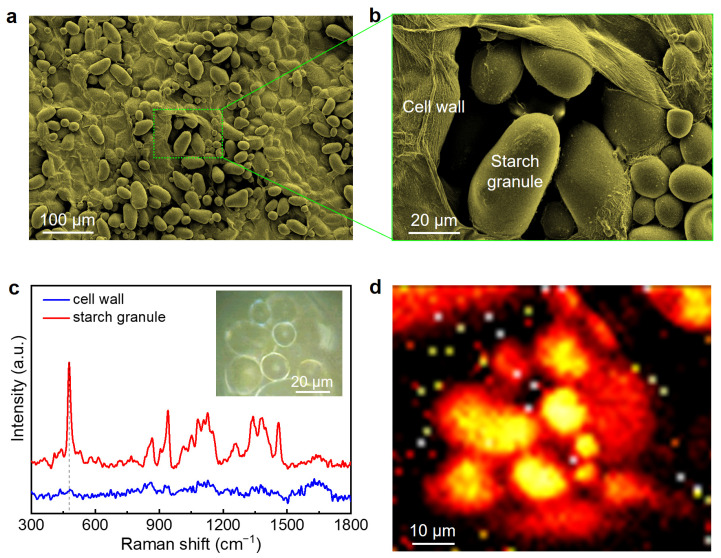
Normal imaging techniques for potato cell characterization. (**a**,**b**) SEM images of potato cells with different magnifications. (**c**) Raman spectra of the potato cell wall (red) and starch granule (blue). Inset: optical image of the sample area for Raman measurement. (**d**) Raman mapping of potato cell sample in (**c**) using the peak intensity of 477 cm^−1^.

Potato cells mainly contain starch granules and cell walls, which makes it a simple plant cell structure for our study. Furthermore, starch granules exist in most plant cells and serve as the carbon storage; they are important starting materials for various industrial applications including biotechnology and biofuel production. The plant cell wall, which is composed of polysaccharides cellulose, hemicellulose, and pectin, acts as a semi-permeable layer that precisely permits the entry of smaller particles and inhibits larger ones. To examine the complete structural information of potato cells, common imaging characterizations such as scanning electron microscopy (SEM) and Raman spectroscopy were performed. To acquire the SEM imaging, potato samples needed to be fully dehydrated via chemical fixation due to the high vacuum chamber. Thus, the natural structure of the potato cells may be altered and structure artifact may be caused. Figure 1a,b shows the typical SEM images of potato cells. As shown in Figure 1b, the starch granules and cell wall were clearly observed. However, the cell wall structure was denaturized and even damaged due to the drying process.

Raman spectroscopy, as one of the noninvasive techniques, yields highly compound specific information for chemical analysis and has great potential for direct imaging. To reveal the intrinsic structure of potato cells, we carried out the Raman measurement on a fresh potato. Figure 1c compares the Raman spectra taken on the starch granules and the cell wall of a fresh potato cell. The starch granules show significant Raman peaks in wavenumber ranging from 300 to 1800 cm^−1^. However, no obvious Raman peaks for the cell wall were observed in the same range of interest. Figure 1d displays the Raman mapping of the same potato sample in the Figure 1c insert and was plotted using a peak intensity of 477 cm^−1^, further confirming that the structure of the cell wall cannot be imaged and that only starch granules were visible. In addition, only 2D imaging can be provided via the Raman measurement; it also takes a long time (~2 h) for the mapping data acquisition. In comparison, the home-made multimodal nonlinear microscopy was capable of providing in vivo 3D structure information of biological samples with a scan depth of up to one millimeter in the period of a few minutes.

### 3.2. Nonlinear Optical Properties of Potato Cells

**Figure 2:**
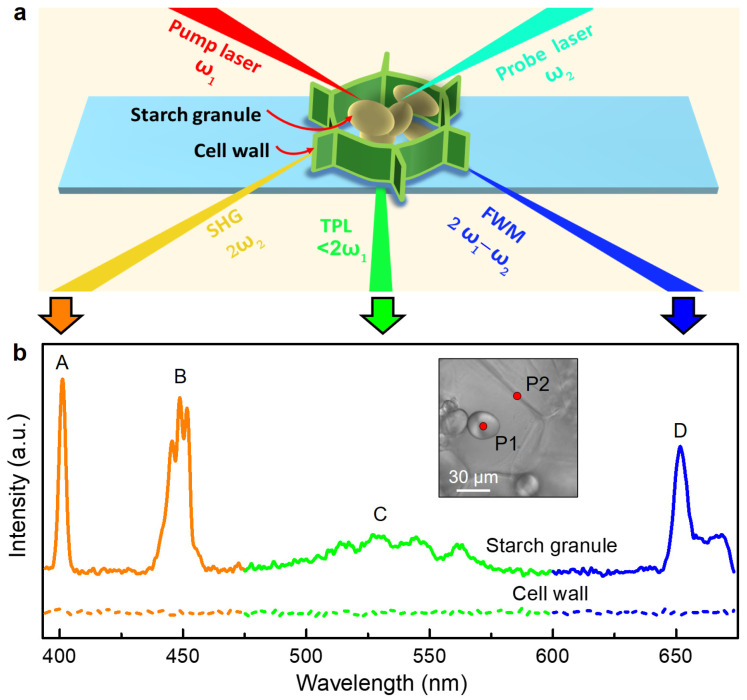
Multiphoton nonlinear optical spectral analyses of potato cells. (**a**) Schematic of the interaction between pump-probe laser beams and a potato cell where the second-order (SHG, TPL) and third-order (FWM) nonlinear optical signals were simultaneously detected. (**b**) Nonlinear optical spectra of starch granule (P1) and cell wall (P2) generated via pump-probe laser beams, where peaks A and B (orange line) represent SHG and SFG signal, respectively; peak C (green line) represents TPL signal, peak D (blue line) represents FWM signal. The measurement points are labeled in the optical image (inset).

To investigate the feasibility of imaging potato cell structures using multimodal nonlinear optical microscopy, we first measured the nonlinear optical spectra of potato cells. As shown in Figure 2a, two fs laser beams were used: one was a pump laser ($\omega_{pump}$) with a wavelength of 800 nm and the other was a probe laser ($\omega_{probe}$) with a broad wavelength ranging between 850 and 1100 nm. The pump-probe laser beams were linearly polarized and collinearly focused onto the fresh potato samples without any pretreatment. Figure 2b shows the nonlinear optical spectra obtained from the potato starch granules (solid curve) and the potato cell walls (short dashed curve). It is obvious that the resulting spectrum of starch granules consisted of four significant signal peaks: a sharp peak at the wavelength of ∼400 nm with Δλ = 7 nm, a strong peak at the wavelength of ∼450 nm with Δλ = 21 nm, a broad weak peak at the wavelength of 500 to 576 nm, and a broad peak at the wavelength of ∼655 nm with Δλ = 12 nm, labeled as “Peak A”, “Peak B”, “Peak C”, and “Peak D”, respectively. Since $\omega_{A}=2\omega_{pump}$, the sharp peak at ~400 nm was the SHG emission from the starch granules, which is consistent with the previous reports [11,26]. “Peak B” originated from the sum-frequency generation (SFG) process because this emission peak was observed at a frequency of $\omega_{B}=\omega_{pump}+\omega_{probe}$. Similarly, “Peak D” was attributed to the FWM process since the peak position corresponded to the frequency of $\omega_{D}=2\omega_{pump}-\omega_{probe}$ [27]. The relatively weak broad peak at ~550 nm originated from the TPL process through interaction with both laser beams. The intrinsic TPL signal of the starch was so weak that it was almost invisible in comparison to the strong SHG. To summarize, under excitation via pump-probe laser beams, both second-order (SHG, SFG, TPL) and third-order (FWM) nonlinear optical behaviors were simultaneously detected from potato starch granules. However, no significant NLO signals from the potato cell walls were observed.

**Figure 3:**
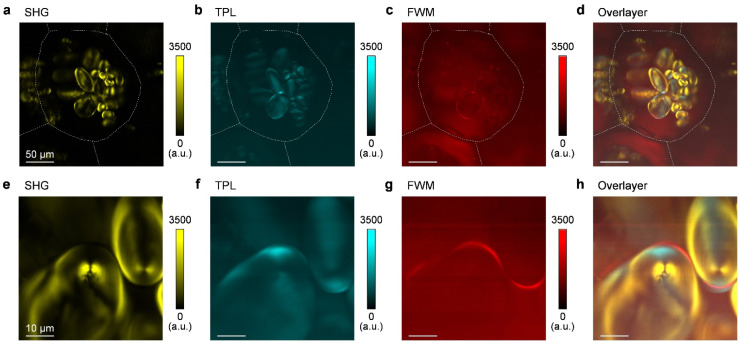
Simultaneous and multimodal nonlinear optical imaging of a potato cell: (**a**) SHG, (**b**) TPL, (**c**) FWM, and (**d**) overlaid SHG-TPL-FWM. The dashed lines in (**a**–**d**) indicate the cell wall of the potato. (**e**,**f**) The magnified (**e**) SHG, (**f**) TPL, (**g**) FWM, and (**h**) overlaid SHG-TPL-FWM images of the starch granule in (**a**–**d**). The scale bars are 50 μm for (**a**–**d**) and 10 μm for (**e**–**h**).

Next, we used nonlinear optical microscopy to perform simultaneous SHG-TPL-FWM optical imaging of potato cells. Figure 3a–d show the SHG, TPL, FWM, and overlaid SHG-TPL-FWM images of a potato cell, respectively. The cell walls were marked with white dotted lines. It was found that only potato starch granules were visible, while no structure information on the potato cell walls was observed. This observation was in accordance with the NLO spectra acquired in Figure 2b. Another interesting observation was that the SHG, TPL, and FWM images can provide different, yet complementary information to each other. Figure 3e–h displays the corresponding SHG, TPL, FWM images with a greater magnification in Figure 3a–c. It can be seen from Figure 3e,f that the distribution of TPL signal in the potato starch grains was complementary to that of SHG. A similar phenomenon was also observed in the collagen whose SHG and TPL signals highly depended on their spatial arrangement [28]. In comparison, FWM imaging (Figure 3g) had a relatively weaker contrast to that of TPL and SHG, with the strongest FWM signal distributed at the edge of the starch granules.

From the above analyses, it can be concluded that in order to realize the imaging of complete potato cells using multimodal nonlinear optical microscopy, an effective labeling approach for cell walls must be developed. In the next section, we investigated the possibility of fs laser-assisted growth of noble metal nanoparticles (NPs) as nanoprobes for cell structure labeling and imaging.

### 3.3. Nonlinear Optical Properties of Fs Laser-Induced Noble Metal NPs

**Figure 4:**
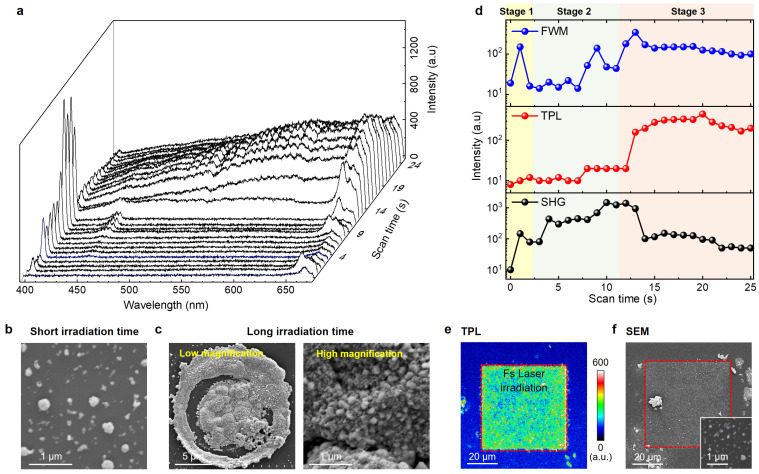
Femtosecond laser-induced silver NPs growth and its nonlinear optical responses. (**a**) Nonlinear optical spectra acquired on fused silica substrate in silver ion solution under a focused fs laser irradiation with different accumulation time. (**b**,**c**) SEM images of (**b**) silver nanoparticles formed with short-time fs laser irradiation and (**c**) silver nanoparticle aggregates formed with a long-time fs laser irradiation. (**d**) The peak intensity of SHG (lower), TPL (middle), and FWM (upper) extracted from (**a**) as a function of fs laser irradiation time. (**e**) TPL mapping of a fused silica substrate in silver ion solution under fs laser scan in a square pattern (red dashed box), (**f**) with the corresponding SEM image, where the red dashed box marks fs laser scan region. Inset in (**f**) shows the enlarged SEM image of square region. As expected, patterned silver NPs were grown.

Previous studies have shown that noble metal ions, such as silver ions (Ag^+^), can be reduced to silver NPs via fs laser scan due to the multi-photon absorption or thermal effect [29,30]. To monitor the noble metal NP formation process and its nonlinear optical properties, we performed in situ, real-time nonlinear optical spectral measurements in a silver ion solution under a focused fs laser irradiation. Figure 4a shows the nonlinear optical spectra acquired on the fused silica substrate in the silver ion solution as a function of fs laser irradiation times. Each spectrum had an accumulation time of 0.2 s. At the beginning, weak SHG signals and a slight background signal measuring around 665 nm were observed since few silver NPs were formed (Stage 1). To confirm this, we performed a SEM imaging study (Figure 4b) where a few silver NPs with an average size of 140 nm were randomly formed on the substrate surface. As the fs laser irradiation time progressed, a significant SHG peak signal centered at ~400 nm, a TPL signal with a broad peak at 475–625 nm, and a broad FWM peak signal at ∼655 nm were observed. Figure 4d reveals the evolution of the nonlinear optical signals of the SHG, TPL, and FWM extracted from Figure 4a. With increasing laser irradiation time, the SHG signal (lower panel, Figure 4d) increased quickly and reached a maximum value at the time of ~10 s, reflecting that many silver nanoparticles were formed on the substrate (Stage 2). However, with further increasing laser irradiation time, the SHG signal decreased by an order of magnitude, which suggests that the silver NPs aggregated into cluster structures (Stage 3). As shown in Figure 4c, large aggregates composed of silver NPs were formed. For TPL (Figure 4d, middle panel), the signal slowly increased at the beginning, reached the maximum at around 15 s, and then maintained a stable and strong value with further laser irradiation. The FWM signal (Figure 4d, upper panel) had a similar trend to that of TPL, which took around 14 s to reach its maximum value. These analyses confirmed that within a short time of fs laser irradiation (just a few seconds), silver NPs with strong nonlinear responses were formed. In addition, the strong nonlinear optical responses were detected in the gold NPs (Figure S1), suggesting that fs laser-induced silver and gold nanostructures were suitable to be used as nanoprobes.

To demonstrate the capability of fs laser-induced noble metal NPs for real-time imaging, we performed the 2D imaging acquisition with a square scan of the fs laser in the noble metal ion solution. Figure 4e shows the TPL image of a fused silica substrate after fs laser scan in the silver ion solution, where the laser scanned area exhibited a uniform and strong nonlinear optical signal. It revealed that the patterned silver NPs with uniform size distribution were formed, as confirmed by the SEM imaging characterization (Figure 4f). Moreover, this experiment demonstrated that in situ silver ion reduction and NP formation can be realized during the nonlinear imaging process, further confirming that fs laser scan induced noble metal NPs can be used as nonlinear optical nanoprobes for nonsignal target imaging.

### 3.4. 3D Construction and Imaging of Potato Cells via Fs Laser Scan in Noble Metal Ion Solution

**Figure 5:**
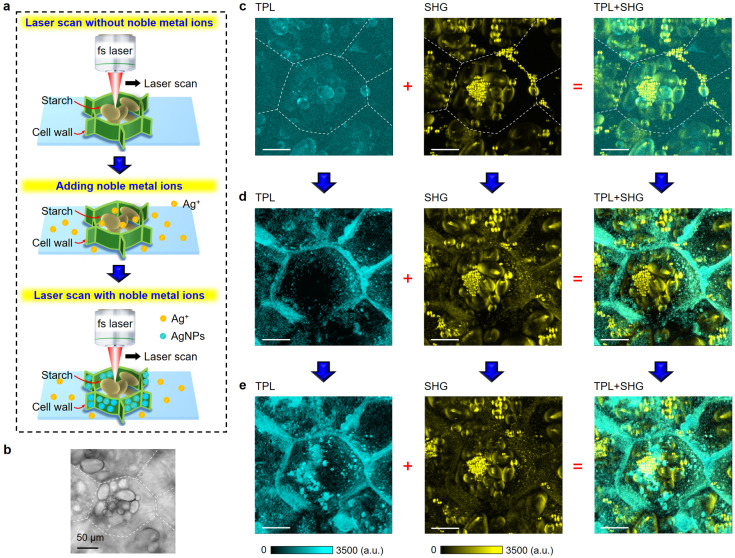
Simultaneous 3D construction and imaging of potato cells in silver ion solution using multimodal nonlinear optical microscopy. (**a**) The schematic of fs laser scan induced imaging and construction of potato cells without (upper panel) and with (middle and lower panels) silver ion solution. (**b**) Optical image of a potato cell. (**c**) 2D Z-stacked TPL (left), SHG (middle), and overlaid TPL-SHG (right) images of the potato cell in (**b**) without adding silver ion solution. The dashed lines in (**b**–**c**) indicate the cell wall of the potato. (**d**) Corresponding TPL (left), SHG (middle), and overlaid TPL-SHG (right) images in (**c**), respectively, taken with fs laser scan only once in silver ion solution. (**e**) Corresponding TPL (left), SHG (middle), and overlaid TPL-SHG (right) images in (**c**) with fs laser scan for the second time in silver ion solution. The scale bar in (**b**–**e**) is 50 μm.

Figure 5a shows the schematic of our proposed approach to one-step 3D cell structural construction and simultaneous potato cell imaging via fs laser scanning of the noble metal ion (Ag^+^ or Au^3+^) solution. It was considered that, during the nonlinear optical imaging process, noble metal ions that crossed the cell wall (middle panel) can be rapidly reduced to NPs via fs laser and selectively anchored onto both sides of the cell wall (lower panel), thus leading to the simultaneous 3D labeling and imaging of complete potato cell structures. To test this idea, two drops (~5 mL) of silver ion solution were added to a thin slice of a freshly cut potato sample (Figure 5b). Then, multimodal 2D (*x-y* plane) nonlinear optical imaging of the potato cells with a scan depth (*z*) of 150 μm was simultaneously acquired. The details of the revolution of the cell wall structure construction with and without adding silver ion solution was also recorded (Supporting Information, Movies S1 and S2). After that, 3D nonlinear optical images of the potato cells were obtained via projecting all the 2D images in the *z* direction (Figure 5c–e).

For comparison, we first collected multimodal 3D nonlinear optical images of potato cells without adding silver ion solution (Figure 5c); all the images including TPL (left), SHG (middle), and the overlaid TPL-SHG (right) only show the information of the starch granules. We also noted that the SHG image offered a better signal-to-noise ratio and a stronger contrast to that of TPL. This was in accordance with our observed results in Figure 2b that starch has much stronger SHG than TPL. With silver ion solution being added (Figure 5d), both TPL and SHG images show the structure information of the cell walls, confirming that silver NPs were formed during the imaging process and were uniformly anchored onto the cell walls. A careful observation further revealed that the TPL image displayed the cell wall structure with a sharp contrast, while the corresponding SHG image showed both the starch grains and the cell wall structure, although with the cell wall having a much weaker contrast than that of TPL. When merging SHG with TPL together, the whole potato cell structure including the starch granules and the cell walls can be clearly imaged, as shown in the overlaid TPL-SHG image in Figure 5d.

To investigate the influence of fs laser scan time on the cell wall structure labeling, the same potato sample in Figure 5b was imaged for a second time (Supporting Information, Movie S3). Figure 5e shows the 3D nonlinear optical imaging acquired with the second implementation of fs laser scanning in the silver ion solution. In comparison to the first scan (Figure 5d), there was no obvious enhancement to the SHG or TPL signal intensity or contract. Thus, a single laser scan was enough for silver ion reduction, NP formation, labeling, and the imaging of cell structures. Moreover, similar results were also observed through performing identical experiments on gold ion solution (Figure S2), further supporting our idea that fs laser-induced noble metal NPs can be used as very good nonlinear optical nanoprobes for in situ and simultaneous plant cell labeling and imaging.

Compared to conventional labeling and imaging methods, there are several advantages of our proposed approach. First, we did not use traditional nonlinear dyes or nanoprobes for labeling, which suffer from photobleaching. Instead, noble metal NPs with no photobleaching effect and with strong NLO properties were used. Second, differing to the conventional cell structure modification and imaging based on noble metal NPs, where noble metal NPs were first fabricated outside the cell and then transported into the cells, we here realize the modification of the cell structure with noble metal NPs directly via fs laser scanning during imaging. Overall, our 3D labeling and imaging approach is one-step, simple, and cost-effective through imaging the cells in the noble metal ion solution without any other treatments.

## 4. Conclusions

In summary, we have developed a simple, one-step approach to 3D plant cell structure labeling and imaging through using noble metal ion-assisted multimodal nonlinear optical microscopy. Instead of fabricating noble metal nanoprobes outside and then transporting them into the cell for labeling/imaging, we realized fs laser-assisted noble metal NP growth and in vivo labeling during the 3D imaging process in a noble metal ion solution. We found that fs laser-induced silver NPs exhibit various excellent nonlinear optical responses including TPL, SHG and FWM. In addition, these silver NPs can be selectively anchored onto the cell walls, thus enabling the simultaneous 3D labeling and nonlinear optical imaging of complete potato cell structures. Similar results have also been observed when substituting silver ions for gold ions. Thus, our method offers a new avenue for facile and in vivo 3D labeling/imaging of plant cells and other cell structures, which is very useful for future biological applications.

## Data Availability

The data that support the findings of this study are available on request from the corresponding author.

## Supplementary Materials

The following supporting information can be downloaded at: https://www.mdpi.com/article/10.3390/nano13192626/s1, Nonlinear optical spectra of fs laser-induced noble metal nanoparticles; simultaneous 3D construction and imaging of potato cells in gold ion solution (PDF); Movie S1: 3D TPL-SHG imaging of potato cells without adding silver ion solution (AVI); Movie S2: 3D labeling and TPL-SHG imaging of potato cells in silver ion solution (1st time fs laser scan) (AVI); Movie S3: 3D labeling and TPL-SHG imaging of potato cells in silver ion solution (2nd time fs laser scan) (AVI); Figure S1: Nonlinear optical responses of noble metal nanoparticles produced via femtosecond laser; Figure S2: Simultaneous 3D construction and nonlinear optical imaging of potato cells in gold ion solution.
