# Supplementary material for: Simultaneous 3D Construction and Imaging of Plant Cells Using Plasmonic Nanoprobe-Assisted Multimodal Nonlinear Optical Microscopy
Source: Nanomaterials (Basel). 2023 Sep 23;13(19):2626. doi: 10.3390/nano13192626 (PMC10574158; doi:10.3390/nano13192626)
Supplement: Supplementary file 1 [file nanomaterials-13-02626-s001.zip › Supporting Information_DL.pdf]

Supporting Information for

Simultaneous 3D Construction and Imaging of Plant Cells Using Plasmonic Nanoprobe Assisted Multimodal Nonlinear Optical Microscopy

*Kun Liu,[1] Yutian Lei,[2] Dawei Li[1,]**

[1] School of Optoelectronic Engineering and Instrumentation Science, Dalian University of Technology, Dalian, Liaoning 116024, China

[2] Department of Civil Engineering, University of Nebraska-Lincoln, Lincoln, NE 68588, United States

* Address correspondence to: dwli@dlut.edu.cn

S1. Nonlinear optical responses of noble metal nanoparticles produced by femtosecond laser

S2. Simultaneous 3D construction and nonlinear optical imaging of potato cells in gold ion solution

S1. Nonlinear optical responses of noble metal nanoparticles produced by femtosecond laser

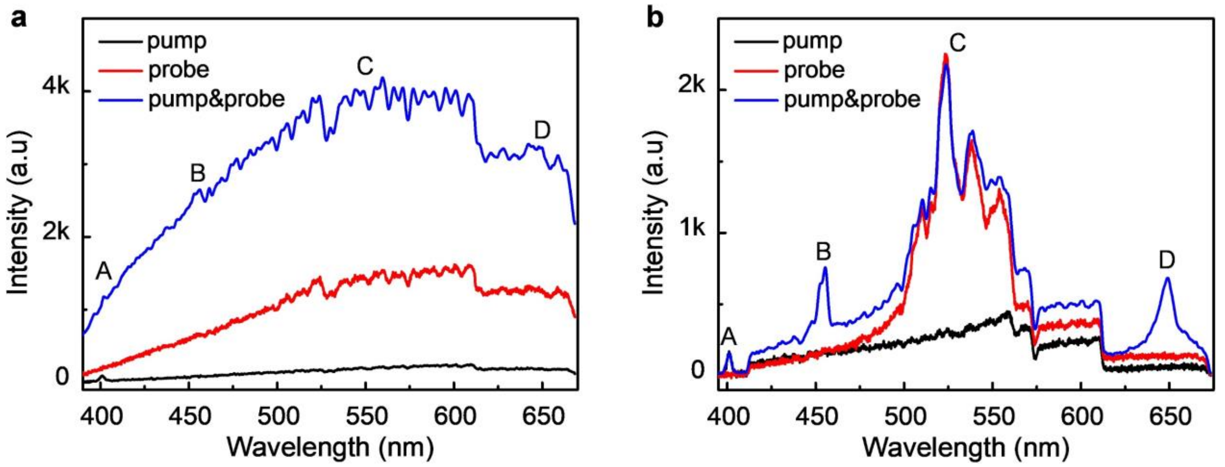

Figure S1. Nonlinear optical spectra of (a) silver NPs and (b) gold NPs on fused silica substrates produced by femtosecond (fs) laser photoreduction of silver and gold ion solution, respectively.

S2. Simultaneous 3D construction and nonlinear optical imaging of potato cells in gold ion solution

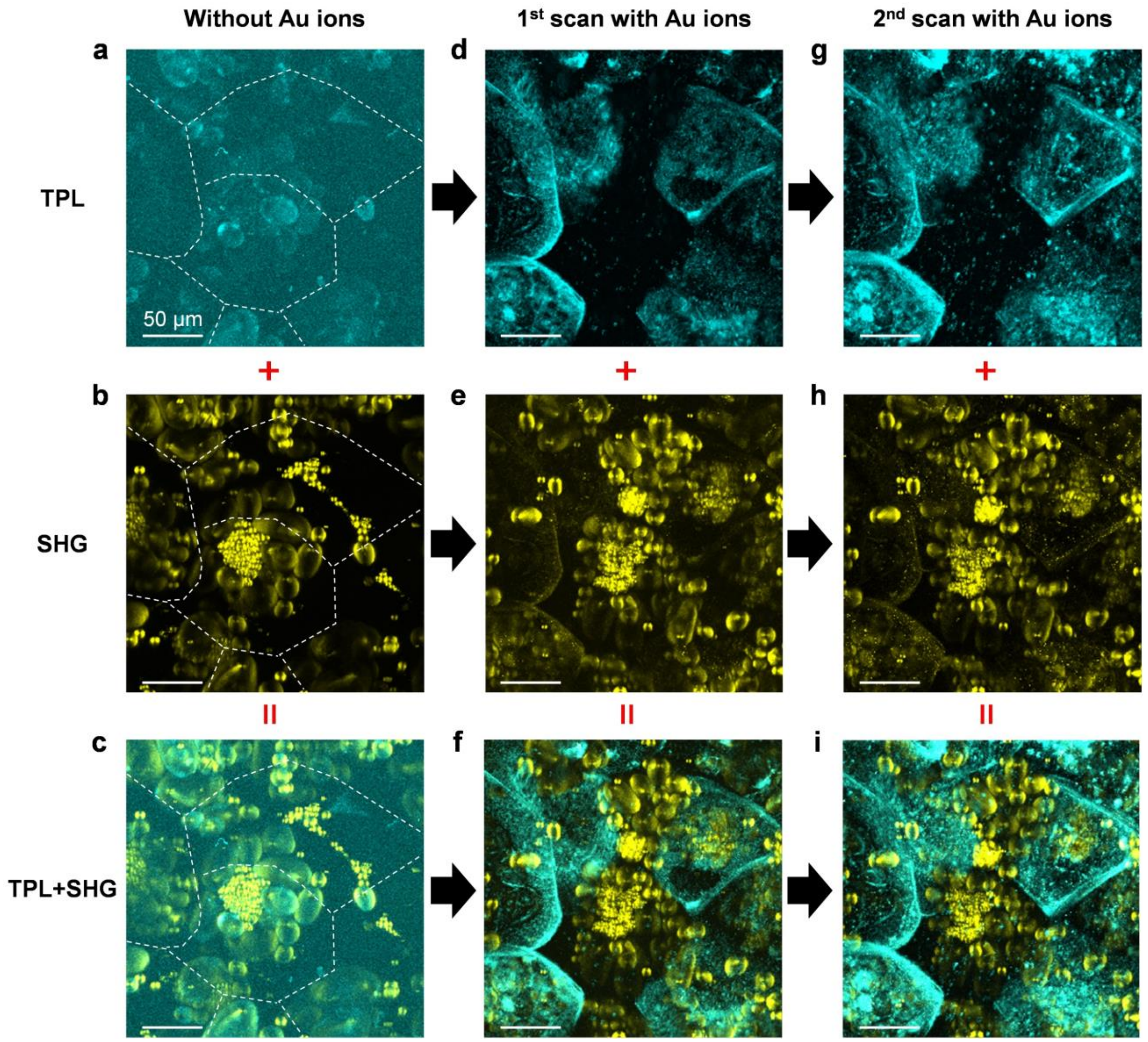

Figure S2. (a-c) 2D Z-stacked (a) TPL, (b) SHG, and (c) overlaid TPL-SHG images of the potato cell without adding gold ion solution. The dashed lines indicate the cell wall of the potato. (d-f) Corresponding TPL, SHG, and overlaid TPL-SHG images in (a-c), respectively, taken with fs laser scan only for one time in gold ion solution. (e) Corresponding TPL, SHG, and overlaid TPL-SHG images in (a-c) with fs laser scan for the second time in the same gold ion solution. The scale bars are 50 μm.
